# (*E*)-3,4,5-Trimeth­oxy-*N*′-[(4-oxo-4*H*-chromen-3-yl)methyl­idene]benzo­hydrazide

**DOI:** 10.1107/S1600536814005753

**Published:** 2014-03-26

**Authors:** Yoshinobu Ishikawa, Kohzoh Watanabe

**Affiliations:** a School of Pharmaceutical Sciences, University of Shizuoka, 52-1 Yada, Suruga-ku, Shizuoka 422-8526, Japan

## Abstract

In the title chromone-tethered benzohydrazide derivative, C_20_H_18_N_2_O_6_, the atoms of the *E*-3-(hydrazonometh­yl)-4*H*-chromen-4-one segment are essentially coplanar, the largest deviation being 0.065 (6) Å. The dihedral angle between this segment and the benzene ring of the tri­meth­oxy­benzene unit is 40.18 (10) Å. In the crystal, the mol­ecule is linked to its inverse-symmetry equivalent by pairs of N—H⋯O hydrogen bonds and C—H⋯π inter­actions. The –CH=N—NH– segment is stacked on the benzene ring of the chromone unit of a translation-related equivalent mol­ecule [centroid–centroid distance = 3.413 (6) Å].

## Related literature   

For the biological activity of related compounds, see: Khan *et al.* (2009 ▶); Tu *et al.* (2013 ▶). For related structures, see: Wang *et al.* (2007 ▶); Qin *et al.* (2009 ▶); Ishikawa *et al.* (2013*a* ▶,*b* ▶,*c* ▶).
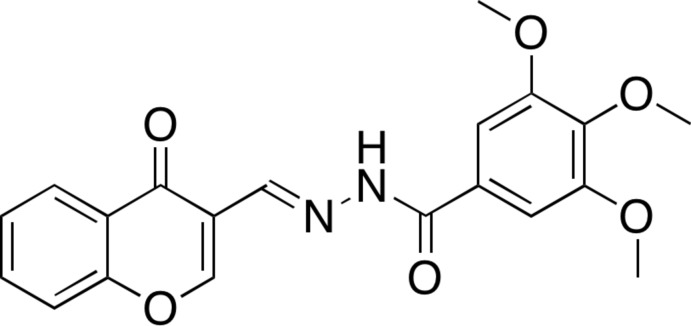


## Experimental   

### 

#### Crystal data  

C_20_H_18_N_2_O_6_
*M* *_r_* = 382.37
Triclinic, 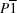

*a* = 6.628 (7) Å
*b* = 11.029 (18) Å
*c* = 12.544 (18) Å
α = 105.34 (12)°
β = 96.94 (10)°
γ = 95.88 (10)°
*V* = 869 (2) Å^3^
*Z* = 2
Mo *K*α radiation
μ = 0.11 mm^−1^
*T* = 100 K
0.40 × 0.18 × 0.12 mm

#### Data collection  

Rigaku AFC-7R diffractometer
4902 measured reflections
3982 independent reflections
1962 reflections with *F* ^2^ > 2σ(*F* ^2^)
*R*_int_ = 0.040
3 standard reflections every 150 reflections intensity decay: 1.9%

#### Refinement  

*R*[*F* ^2^ > 2σ(*F* ^2^)] = 0.068
*wR*(*F* ^2^) = 0.208
*S* = 1.01
3982 reflections
256 parameters
H-atom parameters constrained
Δρ_max_ = 0.30 e Å^−3^
Δρ_min_ = −0.34 e Å^−3^

### 

Data collection: *WinAFC* (Rigaku, 1999 ▶); cell refinement: *WinAFC*; data reduction: *WinAFC*; program(s) used to solve structure: *SHELXS97* (Sheldrick, 2008 ▶); program(s) used to refine structure: *SHELXL97* (Sheldrick, 2008 ▶); molecular graphics: *CrystalStructure* (Rigaku, 2010 ▶); software used to prepare material for publication: *CrystalStructure*.

## Supplementary Material

Crystal structure: contains datablock(s) General, I. DOI: 10.1107/S1600536814005753/zl2582sup1.cif

Structure factors: contains datablock(s) I. DOI: 10.1107/S1600536814005753/zl2582Isup2.hkl

Supporting information file. DOI: 10.1107/S1600536814005753/zl2582Isup3.cml

CCDC reference: 991676

Additional supporting information:  crystallographic information; 3D view; checkCIF report

## Figures and Tables

**Table 1:** Hydrogen-bond geometry (Å, °) *Cg* is the centroid of the C12–C17 ring.

*D*—H⋯*A*	*D*—H	H⋯*A*	*D*⋯*A*	*D*—H⋯*A*
N2—H7⋯O2^i^	0.88	2.10	2.942 (6)	159
C4—H2⋯*Cg* ^i^	0.95	2.97	3.716 (8)	136

Symmetry code: (i) 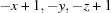.

**Fig. 1.**
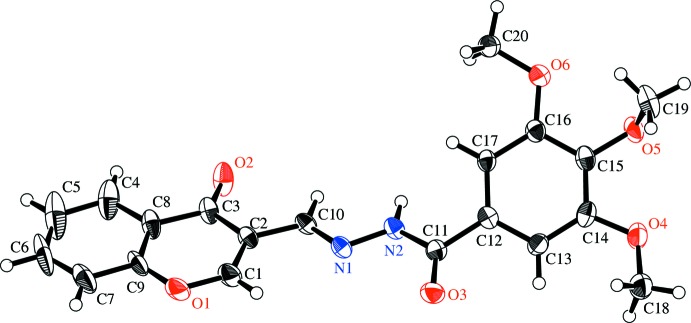
The molecular structure of the title compound, with displacement ellipsoids drawn at the 50% probability level. Hydrogen atoms are shown as small spheres of arbitrary radius.

**Fig. 2.**
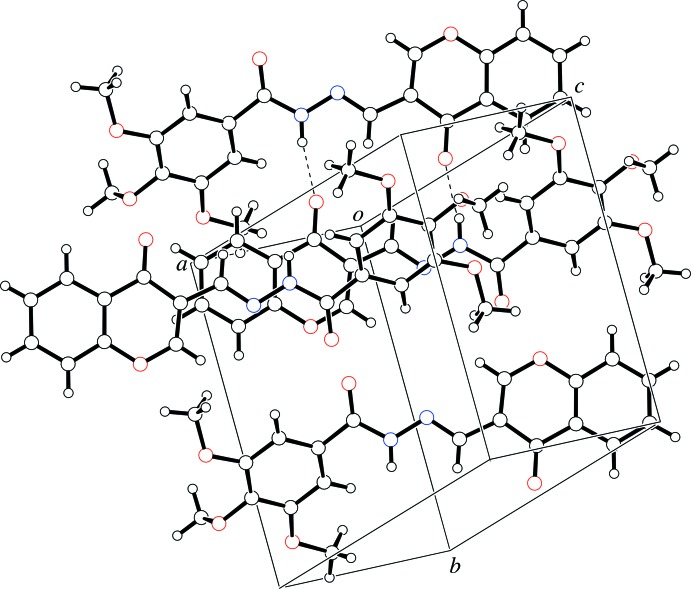
A crystal packing view of the title compound. Intermolecular N–H···O hydrogen bonds are represented by dashed lines.

## supplementary crystallographic information

### 1. Comment

Schiff base derivatives of 3-formyl chromones have attracted much attention due to their biological functions such as enzyme inhibition (Khan *et al.*, 2009; Tu *et al.*, 2013). We herein report the crystal structure of the title compound, which was obtained from the condensation reaction of 3-formylchromone with 3,4,5-trimethoxybenzoylhydrazide in benzene in good yield. The structure (Figure 1) shows that the atoms of the *E*-3-(hydrazonomethyl)-4*H*-chromen-4-one segment are essentially coplanar [the largest deviation = 0.065 (6) Å for C4]. The dihedral angle between this segment and the benzene ring of the trimethoxybenzene unit is 40.18 (10) Å. The carbonyl (C11–O3) group is slightly twisted with respect to this segment [N1–N2–C11–O3 = 12.6 (5)°]. In the crystal, the molecule is linked to its inverse-symmetry equivalent (i: –*x* + 1, –*y*, –*z* + 1) by N–H···O hydrogen bonds, and by C–H···π interaction between the benzene ring of the trimethoxybenzene unit and the C4–H2 atom of the chromone segment [centroid···C4 distance = 3.716 (8) Å]. In addition, the –CH=N–NH– segment is stacked on the benzene ring of the chromone unit of a translation-related equivalent molecule (ii: *x* + 1, *y*, *z*) [centroid–centroid distance = 3.413 (6) Å]. Figure 2 illustrates these intermolecular interactions.

### 2. Experimental

3,4,5-Trimethoxybenzoylhydrazide (1.00 mmol) and 3-formylchromone (1.00 mmol) were dissolved in 25 ml of benzene, and the mixture was refluxed with a Dean-Stark apparatus for 6 h. After cooling, the precipitates were collected, washed with *n*-hexane and dried (yield 75.6%). ^1^H NMR (400 MHz, DMSO-*d*_6_): *δ* = 3.74 (s, 3H), 3.88 (br s, 6H), 7.28 (s, 2H), 7.57 (t, 1H, *J* = 7.6 Hz), 7.75 (d, 1H, *J* = 8.3 Hz), 7.88 (t, 1H, *J* = 7.8 Hz), 8.16 (d, 1H, *J* = 7.8 Hz), 8.67 (s, 1H), 8.87 (s, 1H), 11.82 (s, 1H). DART-MS calcd for [C_20_H_18_N_2_O_6_ + H^+^]: 383.116, found 383.189. Single crystals suitable for X-ray diffraction were obtained by slow evaporation of an acetonitrile solution of the title compound at room temperature.

### 3. Refinement

The C(*sp*^2^)- and N(*sp*^2^)-bound hydrogen atoms were placed in geometrical positions [C–H 0.95 Å, *U*_iso_(H) = 1.2*U*_eq_(C), N–H 0.88 Å, *U*_iso_(H) = 1.2*U*_eq_(N)], and refined using a riding model. Hydrogen atoms of methyl groups were found in a difference Fourier map, and a rotating group model was applied with distance constraint [C–H = 0.98 Å, *U*_iso_(H) = 1.2*U*_eq_(C)].

### Crystal data

**Table:**

C_20_H_18_N_2_O_6_	*Z* = 2
*M**_r_* = 382.37	*F*(000) = 400.00
Triclinic, *P*1	*D*_x_ = 1.461 Mg m^−^^3^
Hall symbol: -P 1	Mo *K*α radiation, λ = 0.71069 Å
*a* = 6.628 (7) Å	Cell parameters from 25 reflections
*b* = 11.029 (18) Å	θ = 15.1–17.5°
*c* = 12.544 (18) Å	µ = 0.11 mm^−^^1^
α = 105.34 (12)°	*T* = 100 K
β = 96.94 (10)°	Plate, yellow
γ = 95.88 (10)°	0.40 × 0.18 × 0.12 mm
*V* = 869 (2) Å^3^	

### Data collection

**Table:**

Rigaku AFC-7R diffractometer	θ_max_ = 27.5°
ω–2θ scans	*h* = −8→4
4902 measured reflections	*k* = −14→14
3982 independent reflections	*l* = −16→16
1962 reflections with *F*^2^ > 2σ(*F*^2^)	3 standard reflections every 150 reflections
*R*_int_ = 0.040	intensity decay: 1.9%

### Refinement

**Table:**

Refinement on *F*^2^	Secondary atom site location: difference Fourier map
*R*[*F*^2^ > 2σ(*F*^2^)] = 0.068	Hydrogen site location: inferred from neighbouring sites
*wR*(*F*^2^) = 0.208	H-atom parameters constrained
*S* = 1.01	*w* = 1/[σ^2^(*F*_o_^2^) + (0.093*P*)^2^ + 0.2643*P*] where *P* = (*F*_o_^2^ + 2*F*_c_^2^)/3
3982 reflections	(Δ/σ)_max_ < 0.001
256 parameters	Δρ_max_ = 0.30 e Å^−^^3^
0 restraints	Δρ_min_ = −0.34 e Å^−^^3^
Primary atom site location: structure-invariant direct methods	

### Special details

**Table:**

Refinement. Refinement was performed using all reflections. The weighted *R*-factor (*wR*) and goodness of fit (*S*) are based on *F*^2^. *R*-factor (gt) are based on *F*. The threshold expression of *F*^2^ > 2.0 σ(*F*^2^) is used only for calculating *R*-factor (gt).

### Fractional atomic coordinates and isotropic or equivalent isotropic displacement parameters (Å^2^)

**Table:**

	*x*	*y*	*z*	*U*_iso_*/*U*_eq_	
O1	1.0097 (4)	0.3621 (3)	0.4260 (3)	0.0470 (8)	
O2	0.7771 (4)	0.0019 (3)	0.4167 (3)	0.0416 (8)	
O3	0.2618 (4)	0.4738 (3)	0.6820 (2)	0.0369 (7)	
O4	−0.3434 (4)	0.4096 (3)	0.8991 (3)	0.0413 (7)	
O5	−0.2570 (4)	0.2233 (3)	0.9857 (2)	0.0357 (7)	
O6	0.0813 (4)	0.1163 (3)	0.9562 (2)	0.0359 (7)	
N1	0.5082 (5)	0.3018 (3)	0.5846 (3)	0.0267 (7)	
N2	0.3513 (5)	0.2739 (3)	0.6410 (3)	0.0266 (7)	
C1	0.8476 (6)	0.3386 (4)	0.4747 (4)	0.0375 (10)	
C2	0.7663 (5)	0.2226 (3)	0.4787 (3)	0.0257 (8)	
C3	0.8540 (6)	0.1112 (4)	0.4232 (3)	0.0298 (9)	
C4	1.1452 (7)	0.0413 (5)	0.3228 (4)	0.0539 (13)	
C5	1.3098 (8)	0.0687 (7)	0.2729 (4)	0.0764 (19)	
C6	1.3754 (7)	0.1940 (7)	0.2766 (4)	0.077 (2)	
C7	1.2771 (7)	0.2920 (6)	0.3295 (4)	0.0602 (15)	
C8	1.0377 (6)	0.1377 (4)	0.3740 (3)	0.0350 (10)	
C9	1.1067 (6)	0.2617 (5)	0.3765 (3)	0.0402 (10)	
C10	0.5918 (6)	0.2068 (3)	0.5368 (3)	0.0267 (8)	
C11	0.2483 (6)	0.3672 (4)	0.6948 (3)	0.0267 (8)	
C12	0.1178 (6)	0.3282 (3)	0.7727 (3)	0.0264 (8)	
C13	−0.0553 (6)	0.3875 (4)	0.7941 (3)	0.0297 (8)	
C14	−0.1749 (5)	0.3547 (4)	0.8687 (3)	0.0305 (9)	
C15	−0.1287 (6)	0.2606 (4)	0.9181 (3)	0.0292 (8)	
C16	0.0466 (6)	0.2031 (4)	0.8991 (3)	0.0277 (8)	
C17	0.1710 (5)	0.2381 (3)	0.8267 (3)	0.0254 (8)	
C18	−0.3580 (6)	0.5322 (4)	0.8866 (4)	0.0409 (10)	
C19	−0.1850 (7)	0.2802 (5)	1.1040 (4)	0.0505 (12)	
C20	0.2601 (6)	0.0549 (4)	0.9390 (4)	0.0399 (10)	
H1	0.7842	0.4090	0.5095	0.0450*	
H2	1.1038	−0.0438	0.3228	0.0647*	
H3	1.3791	0.0024	0.2357	0.0916*	
H4	1.4902	0.2123	0.2420	0.0920*	
H5	1.3246	0.3776	0.3336	0.0723*	
H6	0.5394	0.1242	0.5392	0.0321*	
H7	0.3172	0.1951	0.6423	0.0319*	
H8	−0.0910	0.4496	0.7580	0.0357*	
H9	0.2919	0.2004	0.8144	0.0304*	
H10A	−0.3952	0.5262	0.8071	0.0491*	
H11B	−0.2255	0.5863	0.9158	0.0491*	
H12C	−0.4636	0.5692	0.9281	0.0491*	
H13A	−0.2856	0.2532	1.1473	0.0606*	
H14B	−0.1676	0.3729	1.1201	0.0606*	
H15C	−0.0532	0.2530	1.1245	0.0606*	
H16A	0.2699	−0.0048	0.9843	0.0479*	
H17B	0.3828	0.1189	0.9611	0.0479*	
H18C	0.2499	0.0088	0.8597	0.0479*	

### Atomic displacement parameters (Å^2^)

**Table:**

	*U*^11^	*U*^22^	*U*^33^	*U*^12^	*U*^13^	*U*^23^
O1	0.0324 (16)	0.0546 (18)	0.068 (2)	−0.0022 (14)	0.0109 (15)	0.0423 (16)
O2	0.0325 (15)	0.0253 (14)	0.0578 (18)	−0.0070 (11)	0.0195 (13)	−0.0058 (13)
O3	0.0488 (17)	0.0308 (15)	0.0379 (16)	0.0129 (12)	0.0143 (13)	0.0152 (12)
O4	0.0326 (16)	0.0417 (16)	0.0572 (19)	0.0097 (13)	0.0201 (14)	0.0194 (14)
O5	0.0236 (14)	0.0435 (16)	0.0409 (16)	−0.0044 (12)	0.0095 (12)	0.0147 (13)
O6	0.0299 (14)	0.0417 (15)	0.0433 (16)	0.0056 (12)	0.0098 (12)	0.0221 (13)
N1	0.0302 (17)	0.0241 (15)	0.0243 (16)	−0.0015 (13)	0.0062 (13)	0.0053 (13)
N2	0.0323 (17)	0.0190 (14)	0.0276 (16)	−0.0003 (12)	0.0092 (13)	0.0042 (12)
C1	0.032 (3)	0.036 (2)	0.048 (3)	−0.0045 (17)	0.0081 (18)	0.0208 (19)
C2	0.0240 (18)	0.0249 (18)	0.0260 (19)	−0.0043 (14)	0.0037 (15)	0.0065 (15)
C3	0.0259 (19)	0.032 (2)	0.0251 (19)	−0.0047 (16)	0.0067 (15)	−0.0006 (15)
C4	0.031 (3)	0.072 (3)	0.041 (3)	−0.004 (2)	0.0101 (19)	−0.015 (3)
C5	0.033 (3)	0.135 (6)	0.036 (3)	−0.010 (3)	0.011 (2)	−0.013 (3)
C6	0.024 (3)	0.171 (7)	0.034 (3)	−0.012 (4)	0.009 (2)	0.034 (4)
C7	0.023 (3)	0.123 (5)	0.045 (3)	−0.007 (3)	0.001 (2)	0.051 (3)
C8	0.0203 (18)	0.055 (3)	0.0217 (19)	−0.0055 (17)	0.0030 (15)	0.0023 (18)
C9	0.0204 (19)	0.075 (3)	0.031 (2)	−0.0067 (19)	0.0033 (16)	0.031 (2)
C10	0.0296 (19)	0.0195 (17)	0.0302 (19)	−0.0051 (14)	0.0062 (15)	0.0078 (15)
C11	0.032 (2)	0.0265 (18)	0.0203 (17)	0.0056 (15)	0.0046 (15)	0.0026 (14)
C12	0.0280 (19)	0.0243 (18)	0.0238 (18)	−0.0009 (15)	0.0049 (15)	0.0031 (15)
C13	0.0258 (19)	0.0290 (19)	0.035 (2)	0.0061 (15)	0.0045 (16)	0.0095 (16)
C14	0.0208 (19)	0.0310 (19)	0.035 (2)	0.0027 (15)	0.0042 (16)	0.0013 (16)
C15	0.0244 (19)	0.0296 (19)	0.029 (2)	−0.0046 (15)	0.0051 (15)	0.0033 (16)
C16	0.0220 (18)	0.0296 (19)	0.0296 (19)	−0.0007 (15)	0.0029 (15)	0.0073 (15)
C17	0.0193 (17)	0.0288 (18)	0.0266 (18)	0.0010 (14)	0.0038 (14)	0.0061 (15)
C18	0.036 (3)	0.040 (3)	0.050 (3)	0.0058 (18)	0.0121 (19)	0.0155 (19)
C19	0.045 (3)	0.061 (3)	0.046 (3)	−0.004 (2)	0.022 (2)	0.013 (3)
C20	0.025 (2)	0.048 (3)	0.056 (3)	0.0045 (18)	0.0079 (18)	0.031 (2)

### Geometric parameters (Å, º)

**Table:**

O1—C1	1.335 (6)	C12—C13	1.397 (6)
O1—C9	1.380 (6)	C12—C17	1.394 (6)
O2—C3	1.237 (5)	C13—C14	1.390 (6)
O3—C11	1.224 (5)	C14—C15	1.384 (6)
O4—C14	1.371 (5)	C15—C16	1.396 (6)
O4—C18	1.414 (6)	C16—C17	1.398 (6)
O5—C15	1.378 (5)	N2—H7	0.880
O5—C19	1.448 (6)	C1—H1	0.950
O6—C16	1.360 (6)	C4—H2	0.950
O6—C20	1.434 (5)	C5—H3	0.950
N1—N2	1.380 (5)	C6—H4	0.950
N1—C10	1.278 (5)	C7—H5	0.950
N2—C11	1.367 (5)	C10—H6	0.950
C1—C2	1.352 (6)	C13—H8	0.950
C2—C3	1.457 (6)	C17—H9	0.950
C2—C10	1.459 (6)	C18—H10A	0.980
C3—C8	1.468 (6)	C18—H11B	0.980
C4—C5	1.368 (8)	C18—H12C	0.980
C4—C8	1.399 (7)	C19—H13A	0.980
C5—C6	1.392 (11)	C19—H14B	0.980
C6—C7	1.380 (9)	C19—H15C	0.980
C7—C9	1.387 (7)	C20—H16A	0.980
C8—C9	1.387 (7)	C20—H17B	0.980
C11—C12	1.496 (6)	C20—H18C	0.980
			
O1···C3	2.843 (7)	C10···H8^ii^	3.5772
O2···C1	3.559 (8)	C11···H10A^ii^	2.7721
O2···C4	2.877 (6)	C11···H12C^ii^	3.3916
O2···C10	2.845 (6)	C11···H14B^vii^	3.3148
O3···N1	2.765 (6)	C12···H2^v^	3.1739
O3···C13	2.883 (6)	C12···H14B^vii^	3.1794
O3···C17	3.586 (7)	C13···H14B^vii^	2.7634
O4···O5	2.646 (6)	C14···H11B^vii^	3.4286
O4···C19	3.367 (8)	C14···H14B^vii^	3.5351
O5···O6	2.659 (5)	C15···H2^v^	3.3544
O6···C19	3.081 (7)	C15···H3^v^	3.1886
N1···C1	2.825 (6)	C15···H11B^vii^	2.9753
N2···C17	2.842 (6)	C15···H16A^ix^	3.4488
C1···C7	3.570 (8)	C16···H2^v^	2.8659
C1···C8	2.751 (7)	C16···H3^v^	3.3864
C2···C9	2.777 (6)	C16···H11B^vii^	2.8478
C4···C7	2.790 (10)	C16···H12C^vii^	3.5908
C5···C9	2.753 (9)	C16···H16A^ix^	3.5794
C6···C8	2.769 (7)	C17···H2^v^	2.7705
C10···C11	3.507 (7)	C17···H11B^vii^	3.2523
C12···C15	2.782 (7)	C17···H12C^vii^	3.4633
C13···C16	2.795 (7)	C18···H5^iii^	3.1966
C13···C18	2.856 (7)	C18···H12C^viii^	3.1347
C14···C17	2.780 (6)	C18···H13A^viii^	3.5765
C14···C19	3.272 (8)	C18···H14B^viii^	3.4123
C15···C18	3.586 (8)	C18···H14B^vii^	3.5508
C16···C19	3.122 (7)	C18···H15C^vii^	3.4644
C17···C20	2.821 (7)	C19···H4^xii^	3.0738
O1···O1^i^	3.141 (7)	C19···H8^vii^	3.2579
O1···O3^ii^	3.289 (7)	C19···H11B^vii^	3.0282
O1···O3^iii^	3.119 (6)	C19···H12C^viii^	3.0436
O1···C1^i^	3.201 (8)	C19···H16A^ix^	2.9135
O1···C11^ii^	3.531 (7)	C19···H17B^vi^	3.2570
O2···O2^iv^	3.417 (6)	C19···H18C^ix^	3.3301
O2···N2^v^	2.942 (6)	C20···H3^iv^	3.4296
O2···C3^iv^	3.511 (6)	C20···H13A^ix^	3.3118
O2···C4^iv^	3.411 (8)	C20···H15C^ix^	3.3767
O2···C8^iv^	3.539 (7)	C20···H16A^xiii^	3.2931
O2···C10^v^	3.394 (6)	C20···H17B^xiii^	3.5118
O2···C17^v^	3.552 (7)	H1···O1^i^	2.6152
O3···O1^vi^	3.289 (7)	H1···O3^ii^	3.5039
O3···O1^iii^	3.119 (6)	H1···O3^iii^	3.0140
O3···C1^vi^	3.451 (7)	H1···C1^i^	3.4562
O3···C1^iii^	3.285 (7)	H1···C7^i^	3.4734
O3···C18^ii^	3.252 (7)	H1···C9^i^	3.4959
O3···C19^vii^	3.404 (8)	H1···H1^i^	3.3925
O4···N2^vi^	3.472 (7)	H1···H5^vi^	3.4678
O4···C11^vi^	3.397 (6)	H1···H5^i^	2.8587
O4···C17^vi^	3.449 (6)	H1···H8^ii^	3.0273
O4···C18^viii^	3.491 (7)	H2···O2^iv^	3.1647
O5···C20^vi^	3.433 (6)	H2···O6^v^	3.4165
O5···C20^ix^	3.440 (8)	H2···C3^iv^	3.4444
O6···O6^ix^	3.188 (6)	H2···C10^iv^	3.4588
O6···C4^v^	3.536 (8)	H2···C12^v^	3.1739
O6···C20^ix^	3.402 (7)	H2···C15^v^	3.3544
N1···C7^vi^	3.346 (8)	H2···C16^v^	2.8659
N1···C9^vi^	3.400 (7)	H2···C17^v^	2.7705
N1···C13^ii^	3.530 (7)	H2···H6^iv^	3.1034
N2···O2^v^	2.942 (6)	H2···H7^v^	3.2391
N2···O4^ii^	3.472 (7)	H2···H9^v^	3.0382
N2···C8^vi^	3.579 (7)	H2···H18C^v^	3.2006
N2···C9^vi^	3.475 (7)	H3···O2^ii^	3.2680
C1···O1^i^	3.201 (8)	H3···O5^v^	3.1552
C1···O3^ii^	3.451 (7)	H3···O6^v^	3.5440
C1···O3^iii^	3.285 (7)	H3···C3^ii^	3.5809
C1···C11^ii^	3.517 (7)	H3···C15^v^	3.1886
C2···C6^vi^	3.326 (7)	H3···C16^v^	3.3864
C3···O2^iv^	3.511 (6)	H3···C20^iv^	3.4296
C4···O2^iv^	3.411 (8)	H3···H6^iv^	3.4770
C4···O6^v^	3.536 (8)	H3···H9^iv^	3.2772
C4···C16^v^	3.332 (8)	H3···H16A^x^	3.1271
C4···C17^v^	3.475 (8)	H3···H17B^iv^	3.1628
C5···C10^ii^	3.468 (8)	H3···H18C^iv^	2.8620
C6···C2^ii^	3.326 (7)	H4···O3^i^	3.5084
C6···C10^ii^	3.364 (8)	H4···C1^ii^	3.4026
C7···N1^ii^	3.346 (8)	H4···C2^ii^	3.2626
C7···C10^ii^	3.519 (8)	H4···C3^ii^	3.5766
C8···O2^iv^	3.539 (7)	H4···C19^xi^	3.0738
C8···N2^ii^	3.579 (7)	H4···H10A^iii^	3.2117
C9···N1^ii^	3.400 (7)	H4···H13A^xi^	2.0967
C9···N2^ii^	3.475 (7)	H4···H14B^xi^	3.4738
C10···O2^v^	3.394 (6)	H4···H15C^xi^	3.5609
C10···C5^vi^	3.468 (8)	H4···H16A^x^	3.5157
C10···C6^vi^	3.364 (8)	H4···H17B^x^	3.3622
C10···C7^vi^	3.519 (8)	H4···H18C^iv^	3.2072
C11···O1^vi^	3.531 (7)	H5···O3^i^	3.1009
C11···O4^ii^	3.397 (6)	H5···N1^ii^	3.5731
C11···C1^vi^	3.517 (7)	H5···N1^i^	3.4325
C11···C18^ii^	3.332 (7)	H5···C18^iii^	3.1966
C13···N1^vi^	3.530 (7)	H5···H1^ii^	3.4678
C16···C4^v^	3.332 (8)	H5···H1^i^	2.8587
C17···O2^v^	3.552 (7)	H5···H8^iii^	2.9465
C17···O4^ii^	3.449 (6)	H5···H10A^iii^	2.3551
C17···C4^v^	3.475 (8)	H5···H11B^iii^	3.2546
C18···O3^vi^	3.252 (7)	H5···H15C^x^	3.2923
C18···O4^viii^	3.491 (7)	H6···O2^v^	2.5946
C18···C11^vi^	3.332 (7)	H6···C4^vi^	3.3930
C19···O3^vii^	3.404 (8)	H6···C4^iv^	3.5039
C19···C20^ix^	3.565 (9)	H6···C5^vi^	3.3690
C20···O5^ii^	3.433 (6)	H6···C10^v^	3.5073
C20···O5^ix^	3.440 (8)	H6···H2^iv^	3.1034
C20···O6^ix^	3.402 (7)	H6···H3^iv^	3.4770
C20···C19^ix^	3.565 (9)	H6···H6^v^	2.6279
O1···H5	2.5194	H7···O2^v^	2.1022
O2···H2	2.6183	H7···C3^v^	3.3006
O2···H6	2.5661	H7···C8^vi^	3.5060
O3···H7	3.0489	H7···H2^v^	3.2391
O3···H8	2.6446	H8···O1^iii^	3.5409
O4···H8	2.6652	H8···N1^vi^	3.2075
O4···H14B	3.0194	H8···C1^vi^	3.3969
O6···H9	2.6793	H8···C7^iii^	3.5908
O6···H15C	2.5638	H8···C10^vi^	3.5772
N1···H1	2.4883	H8···C19^vii^	3.2579
N2···H6	2.3661	H8···H1^vi^	3.0273
N2···H9	2.5728	H8···H5^iii^	2.9465
C1···H6	3.2916	H8···H14B^vii^	2.4905
C3···H1	3.2753	H8···H15C^vii^	3.2100
C3···H2	2.6819	H9···O2^v^	3.1049
C3···H6	2.6773	H9···O4^ii^	3.0390
C4···H4	3.2406	H9···O5^ii^	3.4102
C5···H5	3.2771	H9···H2^v^	3.0382
C6···H2	3.2470	H9···H3^iv^	3.2772
C7···H3	3.2695	H9···H12C^vii^	3.5166
C8···H3	3.2596	H10A···O3^vi^	2.5210
C8···H5	3.2841	H10A···N1^vi^	3.1491
C9···H1	3.1828	H10A···N2^vi^	3.1687
C9···H2	3.2541	H10A···C6^iii^	3.5086
C9···H4	3.2311	H10A···C7^iii^	3.0639
C10···H1	2.5777	H10A···C11^vi^	2.7721
C10···H7	2.3883	H10A···H4^iii^	3.2117
C11···H8	2.6427	H10A···H5^iii^	2.3551
C11···H9	2.6799	H10A···H13A^viii^	3.3663
C12···H7	2.4955	H10A···H14B^viii^	3.3514
C13···H9	3.2749	H10A···H15C^vii^	3.5062
C13···H10A	2.8471	H11B···O5^vii^	3.5301
C13···H11B	2.7500	H11B···O6^vii^	3.2335
C14···H10A	2.7151	H11B···C14^vii^	3.4286
C14···H11B	2.5313	H11B···C15^vii^	2.9753
C14···H12C	3.1860	H11B···C16^vii^	2.8478
C14···H14B	3.1008	H11B···C17^vii^	3.2523
C15···H8	3.2665	H11B···C19^vii^	3.0282
C15···H9	3.2713	H11B···H5^iii^	3.2546
C15···H13A	3.1908	H11B···H14B^vii^	2.7101
C15···H14B	2.5665	H11B···H15C^vii^	2.6154
C15···H15C	2.6025	H11B···H17B^vii^	3.5313
C16···H14B	3.4580	H12C···O3^vi^	3.2371
C16···H15C	2.9045	H12C···O4^viii^	2.6180
C16···H16A	3.1935	H12C···O5^viii^	3.1429
C16···H17B	2.6111	H12C···C11^vi^	3.3916
C16···H18C	2.6155	H12C···C16^vii^	3.5908
C17···H7	2.5638	H12C···C17^vii^	3.4633
C17···H8	3.2767	H12C···C18^viii^	3.1347
C17···H17B	2.7416	H12C···C19^viii^	3.0436
C17···H18C	2.7583	H12C···H9^vii^	3.5166
C18···H8	2.6086	H12C···H12C^viii^	2.7091
C20···H9	2.5292	H12C···H13A^viii^	2.9594
H1···H6	3.5152	H12C···H14B^viii^	2.6299
H2···H3	2.3168	H12C···H17B^vii^	3.3110
H3···H4	2.3338	H13A···O3^vii^	3.1758
H4···H5	2.3358	H13A···C6^xii^	3.0406
H6···H7	2.1478	H13A···C18^viii^	3.5765
H7···H9	2.1713	H13A···C20^ix^	3.3118
H8···H10A	2.3516	H13A···H4^xii^	2.0967
H8···H11B	2.4712	H13A···H10A^viii^	3.3663
H8···H12C	3.5828	H13A···H12C^viii^	2.9594
H9···H16A	3.4997	H13A···H16A^ix^	2.8195
H9···H17B	2.2971	H13A···H17B^vi^	2.9562
H9···H18C	2.3277	H13A···H18C^ix^	2.9032
O1···H1^i^	2.6152	H14B···O3^vii^	2.7891
O1···H8^iii^	3.5409	H14B···C11^vii^	3.3148
O2···H2^iv^	3.1647	H14B···C12^vii^	3.1794
O2···H3^vi^	3.2680	H14B···C13^vii^	2.7634
O2···H6^v^	2.5946	H14B···C14^vii^	3.5351
O2···H7^v^	2.1022	H14B···C18^viii^	3.4123
O2···H9^v^	3.1049	H14B···C18^vii^	3.5508
O2···H18C^v^	3.4209	H14B···H4^xii^	3.4738
O3···H1^vi^	3.5039	H14B···H8^vii^	2.4905
O3···H1^iii^	3.0140	H14B···H10A^viii^	3.3514
O3···H4^i^	3.5084	H14B···H11B^vii^	2.7101
O3···H5^i^	3.1009	H14B···H12C^viii^	2.6299
O3···H10A^ii^	2.5210	H15C···C6^xiv^	3.4441
O3···H12C^ii^	3.2371	H15C···C7^xiv^	3.0743
O3···H13A^vii^	3.1758	H15C···C9^xiv^	3.1835
O3···H14B^vii^	2.7891	H15C···C18^vii^	3.4644
O4···H9^vi^	3.0390	H15C···C20^ix^	3.3767
O4···H12C^viii^	2.6180	H15C···H4^xii^	3.5609
O5···H3^v^	3.1552	H15C···H5^xiv^	3.2923
O5···H9^vi^	3.4102	H15C···H8^vii^	3.2100
O5···H11B^vii^	3.5301	H15C···H10A^vii^	3.5062
O5···H12C^viii^	3.1429	H15C···H11B^vii^	2.6154
O5···H16A^ix^	2.5300	H15C···H16A^ix^	2.8571
O5···H17B^vi^	2.4880	H15C···H18C^ix^	3.1098
O5···H18C^ix^	3.5978	H16A···O5^ix^	2.5300
O6···H2^v^	3.4165	H16A···O6^ix^	2.7939
O6···H3^v^	3.5440	H16A···C5^xiv^	3.4640
O6···H11B^vii^	3.2335	H16A···C15^ix^	3.4488
O6···H16A^ix^	2.7939	H16A···C16^ix^	3.5794
N1···H5^vi^	3.5731	H16A···C19^ix^	2.9135
N1···H5^i^	3.4325	H16A···C20^xiii^	3.2931
N1···H8^ii^	3.2075	H16A···H3^xiv^	3.1271
N1···H10A^ii^	3.1491	H16A···H4^xiv^	3.5157
N2···H10A^ii^	3.1687	H16A···H13A^ix^	2.8195
C1···H1^i^	3.4562	H16A···H15C^ix^	2.8571
C1···H4^vi^	3.4026	H16A···H16A^xiii^	3.0153
C1···H8^ii^	3.3969	H16A···H17B^xiii^	2.8319
C2···H4^vi^	3.2626	H16A···H18C^xiii^	3.5396
C3···H2^iv^	3.4444	H17B···O5^ii^	2.4880
C3···H3^vi^	3.5809	H17B···C19^ii^	3.2570
C3···H4^vi^	3.5766	H17B···C20^xiii^	3.5118
C3···H7^v^	3.3006	H17B···H3^iv^	3.1628
C3···H18C^v^	3.4000	H17B···H4^xiv^	3.3622
C4···H6^ii^	3.3930	H17B···H11B^vii^	3.5313
C4···H6^iv^	3.5039	H17B···H12C^vii^	3.3110
C4···H18C^v^	3.1614	H17B···H13A^ii^	2.9562
C5···H6^ii^	3.3690	H17B···H16A^xiii^	2.8319
C5···H16A^x^	3.4640	H17B···H17B^xiii^	3.4741
C5···H18C^iv^	3.5979	H18C···O2^v^	3.4209
C6···H10A^iii^	3.5086	H18C···O5^ix^	3.5978
C6···H13A^xi^	3.0406	H18C···C3^v^	3.4000
C6···H15C^x^	3.4441	H18C···C4^v^	3.1614
C7···H1^i^	3.4734	H18C···C5^iv^	3.5979
C7···H8^iii^	3.5908	H18C···C8^v^	3.2174
C7···H10A^iii^	3.0639	H18C···C19^ix^	3.3301
C7···H15C^x^	3.0743	H18C···H2^v^	3.2006
C8···H7^ii^	3.5060	H18C···H3^iv^	2.8620
C8···H18C^v^	3.2174	H18C···H4^iv^	3.2072
C9···H1^i^	3.4959	H18C···H13A^ix^	2.9032
C9···H15C^x^	3.1835	H18C···H15C^ix^	3.1098
C10···H2^iv^	3.4588	H18C···H16A^xiii^	3.5396
C10···H6^v^	3.5073		
			
C1—O1—C9	118.5 (4)	C15—C16—C17	119.5 (4)
C14—O4—C18	117.6 (4)	C12—C17—C16	120.0 (4)
C15—O5—C19	113.4 (3)	N1—N2—H7	119.645
C16—O6—C20	116.6 (4)	C11—N2—H7	119.637
N2—N1—C10	115.0 (3)	O1—C1—H1	117.355
N1—N2—C11	120.7 (3)	C2—C1—H1	117.346
O1—C1—C2	125.3 (4)	C5—C4—H2	119.811
C1—C2—C3	119.3 (4)	C8—C4—H2	119.804
C1—C2—C10	121.3 (4)	C4—C5—H3	120.079
C3—C2—C10	119.4 (4)	C6—C5—H3	120.082
O2—C3—C2	122.3 (4)	C5—C6—H4	119.339
O2—C3—C8	122.5 (4)	C7—C6—H4	119.343
C2—C3—C8	115.3 (4)	C6—C7—H5	121.057
C5—C4—C8	120.4 (5)	C9—C7—H5	121.046
C4—C5—C6	119.8 (6)	N1—C10—H6	119.366
C5—C6—C7	121.3 (5)	C2—C10—H6	119.364
C6—C7—C9	117.9 (6)	C12—C13—H8	120.275
C3—C8—C4	122.0 (4)	C14—C13—H8	120.278
C3—C8—C9	119.5 (4)	C12—C17—H9	119.980
C4—C8—C9	118.5 (4)	C16—C17—H9	119.986
O1—C9—C7	116.1 (5)	O4—C18—H10A	109.468
O1—C9—C8	121.9 (4)	O4—C18—H11B	109.472
C7—C9—C8	122.0 (5)	O4—C18—H12C	109.474
N1—C10—C2	121.3 (4)	H10A—C18—H11B	109.465
O3—C11—N2	123.6 (4)	H10A—C18—H12C	109.472
O3—C11—C12	122.4 (4)	H11B—C18—H12C	109.476
N2—C11—C12	114.0 (4)	O5—C19—H13A	109.472
C11—C12—C13	118.8 (4)	O5—C19—H14B	109.472
C11—C12—C17	121.0 (4)	O5—C19—H15C	109.473
C13—C12—C17	120.1 (4)	H13A—C19—H14B	109.470
C12—C13—C14	119.4 (4)	H13A—C19—H15C	109.473
O4—C14—C13	124.6 (4)	H14B—C19—H15C	109.467
O4—C14—C15	114.8 (4)	O6—C20—H16A	109.471
C13—C14—C15	120.6 (4)	O6—C20—H17B	109.473
O5—C15—C14	119.8 (4)	O6—C20—H18C	109.471
O5—C15—C16	120.0 (4)	H16A—C20—H17B	109.473
C14—C15—C16	120.2 (4)	H16A—C20—H18C	109.472
O6—C16—C15	114.9 (4)	H17B—C20—H18C	109.467
O6—C16—C17	125.6 (4)		
			
C1—O1—C9—C7	−178.5 (3)	H2—C4—C5—H3	2.5
C1—O1—C9—C8	1.5 (5)	H2—C4—C8—C3	−3.3
C9—O1—C1—C2	−1.1 (6)	H2—C4—C8—C9	177.5
C9—O1—C1—H1	178.9	C4—C5—C6—C7	−0.3 (7)
C14—O4—C18—H10A	74.4	C4—C5—C6—H4	179.7
C14—O4—C18—H11B	−45.6	H3—C5—C6—C7	179.7
C14—O4—C18—H12C	−165.6	H3—C5—C6—H4	−0.3
C18—O4—C14—C13	−22.6 (5)	C5—C6—C7—C9	−1.7 (7)
C18—O4—C14—C15	157.7 (3)	C5—C6—C7—H5	178.3
C15—O5—C19—H13A	177.4	H4—C6—C7—C9	178.3
C15—O5—C19—H14B	57.4	H4—C6—C7—H5	−1.7
C15—O5—C19—H15C	−62.6	C6—C7—C9—O1	−178.3 (4)
C19—O5—C15—C14	−99.4 (4)	C6—C7—C9—C8	1.6 (6)
C19—O5—C15—C16	80.8 (4)	H5—C7—C9—O1	1.7
C16—O6—C20—H16A	179.7	H5—C7—C9—C8	−178.4
C16—O6—C20—H17B	59.7	C3—C8—C9—O1	1.2 (5)
C16—O6—C20—H18C	−60.3	C3—C8—C9—C7	−178.8 (3)
C20—O6—C16—C15	180.0 (3)	C4—C8—C9—O1	−179.6 (3)
C20—O6—C16—C17	−0.7 (4)	C4—C8—C9—C7	0.4 (6)
N2—N1—C10—C2	−176.9 (3)	O3—C11—C12—C13	28.2 (5)
N2—N1—C10—H6	3.1	O3—C11—C12—C17	−149.3 (3)
C10—N1—N2—C11	−179.5 (3)	N2—C11—C12—C13	−152.5 (3)
C10—N1—N2—H7	0.5	N2—C11—C12—C17	30.0 (4)
N1—N2—C11—O3	12.6 (5)	C11—C12—C13—C14	−178.2 (3)
N1—N2—C11—C12	−166.8 (3)	C11—C12—C13—H8	1.8
H7—N2—C11—O3	−167.4	C11—C12—C17—C16	−180.0 (3)
H7—N2—C11—C12	13.2	C11—C12—C17—H9	0.0
O1—C1—C2—C3	−2.1 (6)	C13—C12—C17—C16	2.6 (4)
O1—C1—C2—C10	178.3 (3)	C13—C12—C17—H9	−177.4
H1—C1—C2—C3	177.9	C17—C12—C13—C14	−0.7 (4)
H1—C1—C2—C10	−1.7	C17—C12—C13—H8	179.3
C1—C2—C3—O2	−174.5 (3)	C12—C13—C14—O4	177.6 (3)
C1—C2—C3—C8	4.5 (5)	C12—C13—C14—C15	−2.7 (5)
C1—C2—C10—N1	1.0 (5)	H8—C13—C14—O4	−2.4
C1—C2—C10—H6	−179.0	H8—C13—C14—C15	177.3
C3—C2—C10—N1	−178.6 (3)	O4—C14—C15—O5	4.2 (4)
C3—C2—C10—H6	1.4	O4—C14—C15—C16	−176.0 (3)
C10—C2—C3—O2	5.1 (5)	C13—C14—C15—O5	−175.5 (3)
C10—C2—C3—C8	−175.9 (3)	C13—C14—C15—C16	4.2 (5)
O2—C3—C8—C4	−4.2 (5)	O5—C15—C16—O6	−3.2 (4)
O2—C3—C8—C9	175.0 (3)	O5—C15—C16—C17	177.4 (3)
C2—C3—C8—C4	176.7 (3)	C14—C15—C16—O6	177.0 (3)
C2—C3—C8—C9	−4.1 (5)	C14—C15—C16—C17	−2.3 (5)
C5—C4—C8—C3	176.7 (4)	O6—C16—C17—C12	179.7 (3)
C5—C4—C8—C9	−2.5 (6)	O6—C16—C17—H9	−0.3
C8—C4—C5—C6	2.4 (7)	C15—C16—C17—C12	−1.0 (4)
C8—C4—C5—H3	−177.6	C15—C16—C17—H9	179.0
H2—C4—C5—C6	−177.6		

Symmetry codes: (i) −*x*+2, −*y*+1, −*z*+1; (ii) *x*+1, *y*, *z*; (iii) −*x*+1, −*y*+1, −*z*+1; (iv) −*x*+2, −*y*, −*z*+1; (v) −*x*+1, −*y*, −*z*+1; (vi) *x*−1, *y*, *z*; (vii) −*x*, −*y*+1, −*z*+2; (viii) −*x*−1, −*y*+1, −*z*+2; (ix) −*x*, −*y*, −*z*+2; (x) *x*+1, *y*, *z*−1; (xi) *x*+2, *y*, *z*−1; (xii) *x*−2, *y*, *z*+1; (xiii) −*x*+1, −*y*, −*z*+2; (xiv) *x*−1, *y*, *z*+1.

### Hydrogen-bond geometry (Å, º)

Cg is the centroid of the C12–C17 ring.

**Table:**

*D*—H···*A*	*D*—H	H···*A*	*D*···*A*	*D*—H···*A*
N2—H7···O2^v^	0.88	2.10	2.942 (6)	159
C4—H2···*Cg*^v^	0.95	2.97	3.716 (8)	136

Symmetry code: (v) −*x*+1, −*y*, −*z*+1.

## References

[bb1] Ishikawa, Y. & Motohashi, Y. (2013*a*). *Acta Cryst.* E**69**, o1225. 10.1107/S1600536813018072 PMC3793730 24109317

[bb2] Ishikawa, Y. & Motohashi, Y. (2013*b*). *Acta Cryst.* E**69**, o1226. 10.1107/S1600536813018084 PMC3793731 24109318

[bb3] Ishikawa, Y. & Motohashi, Y. (2013*c*). *Acta Cryst.* E**69**, o1448. 10.1107/S160053681302285X PMC3884411 24427075

[bb4] Khan, K. M., Ambreen, N., Hussain, S., Perveen, S. & Choudhary, M. I. (2009). *Bioorg. Med. Chem.* **17**, 2983–2988. 10.1016/j.bmc.2009.03.020 19329330

[bb5] Qin, D. D., Qi, G. F., Yang, Z. Y., Wu, J. C. & Liu, Y. C. (2009). *J. Fluoresc.* **19**, 409–418. 10.1007/s10895-008-0427-x 18937060

[bb6] Rigaku (1999). *WinAFC Diffractometer Control Software* Rigaku Corporation, Tokyo, Japan.

[bb7] Rigaku (2010). *CrystalStructure* Rigaku Corporation, Tokyo, Japan.

[bb8] Sheldrick, G. M. (2008). *Acta Cryst.* A**64**, 112–122. 10.1107/S0108767307043930 18156677

[bb9] Tu, Q. D., Li, D., Sun, Y., Han, X. Y., Yi, F., Sha, Y., Ren, Y. L., Ding, M. W., Feng, L. L. & Wan, J. (2013). *Bioorg. Med. Chem.* **21**, 2826–2831. 10.1016/j.bmc.2013.04.003 23623712

[bb10] Wang, B. D., Yang, Z. Y., Crewdson, P. & Wang, D. Q. (2007). *J. Inorg. Biochem.* **101**, 1492–1504. 10.1016/j.jinorgbio.2007.04.007 17692381

